# Common genetic variants do not predict recurrent events in coronary heart disease patients

**DOI:** 10.1186/s12872-022-02520-0

**Published:** 2022-03-09

**Authors:** P. L. Thompson, J. Hui, J. Beilby, L. J. Palmer, G. F. Watts, M. J. West, A. Kirby, S. Marschner, R. J. Simes, D. R. Sullivan, H. D. White, R. Stewart, A. M. Tonkin

**Affiliations:** 1 Heart and Vascular Research Institute, Harry Perkins Institute of Medical Research, Faculty of Health and Medical Sciences, Sir Charles Gairdner Hospital, University of Western Australia, Hospital Ave, Perth, Nedlands, WA 6009 Australia; 2 Health Department of Western Australia, PathWest, Perth, Australia; 3 School of Population and Global Health, University of Western Australia, Perth, Australia; 4 School of Biomedical Sciences, University of Western Australia, Perth, Australia; 5 School of Public Health, University of Adelaide, Adelaide, Australia; 6 Faculty of Health and Medical Sciences, University of Western Australia, Perth, Australia; 7 Faculty of Medicine and Biomedical Sciences, University of Queensland, Brisbane, Australia; 8 NHMRC Clinical Trials Centre, University of Sydney, Sydney, Australia; 9 Department of Chemical Pathology, Royal Prince Alfred Hospital, Sydney, Australia; 10 Green Lane Cardiovascular Service, Auckland City Hospital, Auckland, New Zealand; 11 School of Public Health and Preventive Medicine, Monash University, Melbourne, Australia

**Keywords:** Genome-wide association studies, GWAS, Genetic variant, Coronary heart disease, LIPID study, Genetic risk scores, Polygenic risk scores, Omnigenic risk scores, Lack of prediction

## Abstract

**Background:**

It is unclear whether genetic variants identified from single nucleotide polymorphisms (SNPs) strongly associated with coronary heart disease (CHD) in genome-wide association studies (GWAS), or a genetic risk score (GRS) derived from them, can help stratify risk of recurrent events in patients with CHD.

**Methods:**

Study subjects were enrolled at the close-out of the LIPID randomised controlled trial of pravastatin vs placebo. Entry to the trial had required a history of acute coronary syndrome 3–36 months previously, and patients were in the trial for a mean of 36 months. Patients who consented to a blood sample were genotyped with a custom designed array chip with SNPs chosen from known CHD-associated loci identified in previous GWAS. We evaluated outcomes in these patients over the following 10 years.

**Results:**

Over the 10-year follow-up of the cohort of 4932 patients, 1558 deaths, 898 cardiovascular deaths, 727 CHD deaths and 375 cancer deaths occurred. There were no significant associations between individual SNPs and outcomes before or after adjustment for confounding variables and for multiple testing. A previously validated 27 SNP GRS derived from SNPs with the strongest associations with CHD also did not show any independent association with recurrent major cardiovascular events.

**Conclusions:**

Genetic variants based on individual single nucleotide polymorphisms strongly associated with coronary heart disease in genome wide association studies or an abbreviated genetic risk score derived from them did not help risk profiling in this well-characterised cohort with 10-year follow-up. Other approaches will be needed to incorporate genetic profiling into clinically relevant stratification of long-term risk of recurrent events in CHD patients.

**Supplementary Information:**

The online version contains supplementary material available at 10.1186/s12872-022-02520-0.

## Introduction

Genome-wide association studies (GWAS) have identified large numbers of loci reliably associated with prevalent CHD [1]. Genetic risk scores (GRS) derived from SNPs which had the strongest associations with CHD improved the prediction of first events [2], but attempts to use a GRS for genetic profiling to predict recurrent events in established CHD have yielded conflicting findings. This may be because they have been statistically underpowered with short-term periods of follow-up [3]. While polygenic risk scores (PRS) including thousands of SNPs have been shown to assist with risk stratification, their value in clinical application remains uncertain [4]. Therefore, in the present study, we tested whether individual SNPs or an abbreviated, previously validated GRS derived from SNPs known to be strongly associated with CHD could be applied clinically to predict long-term cardiovascular outcomes.

## Methods

### The LIPID genetic cohort

The LIPID (Long-term Intervention with Pravastatin in Ischaemic Disease) trial (Trial Registration ACTRN12616000535471) comparing pravastatin 40 mg per day with placebo was conducted between 1992 and 1998 in 9014 patients. Entry to the trial required a history of acute coronary syndrome (ACS) 3–36 months previously, and patients were in the trial for a mean of 36 months. The results have been reported previously [5]. The cohort was followed for a total of 10 years from the close-out of the randomised controlled trial (RCT) [6, 7].

The LIPID Genetic cohort is described in Table 1. It included those patients alive at the end of the RCT in 1998, who had also given consent for collection of a blood sample and had high-quality DNA extracted. Whole blood was not available to enable DNA extraction from samples obtained at the time of patient randomisation. The LIPID Genetic cohort totalled 4932 patients. All fatal events were analysed in the 10 years of follow-up between 1997 and 2006, and all coronary events (fatal and non-fatal) in the first two years of cohort follow-up.

**Table 1:** Numbers of patients who were randomised, survived to end of the LIPID Trial, and were included in the Genetic Cohort study (n = 4932) and events that occurred in the Genetic Cohort (shown in bold in the Table) when followed for 10 years from the end of the trial

	Pravastatin	Placebo	Total
Randomised	4512	4502	9014
Survived to end of RCT (mean 6.1 years)	4014	3868	7882
Consented to blood sample, high quality DNA extracted	2524	2408	**4932** **(LIPID Genetic cohort)**
*LIPID Genetic Cohort (n* = *4932) followed for 10 years from end of RCT*			
All-cause deaths	792 (31.4%)	766 (31.8%)	1558 (31.6%)
Cardiovascular deaths	455 (18.0%)	443 (18.4%)	898 (18.2%)
Coronary heart disease deaths	368 (14.6%)	359 (14.9%)	727 (14.7%)
Cancer deaths	192 (7.6%)	183 (7.6%)	375 (7.6%)

### DNA extraction

DNA was extracted from whole blood samples from consenting patients at their close-out visit and stored at − 80 °C. The reasons for exclusion from the Genetic cohort included death during the trial (n = 1132), lack of consent for DNA extraction at close-out or DNA samples not of suitable quality for analysis (n = 2950).

### Exploration and selection of SNPs

A literature review was undertaken using English language reports in PubMed to select SNPs for further exploration and was based on (1) SNPs with a significance of *p* < 5 × 10^−8^ in published GWAS reports of cardiovascular disease and (2) SNPs from known atherothrombotic pathways and other pathways related to rhythm and conduction disturbances, left ventricular dysfunction/cardiac failure and statin responsiveness. A custom designed Illumina GoldGate array of 384 SNPs with minor allele frequency (MAF) > 1% in European populations was used in this study (See Additional file 1: Table S1).

### Exploration of a previously derived GRS

We explored the predictive value of a GRS derived by Mega et al. [8] This required an additional five SNPS to be included after amplification using Taq Man probe assays. In our testing of this GRS, we created a score for each patient by summing the number of risk alleles for each SNP weighted by the log of the ORs used by Mega et al. [8]. We created an unadjusted model and also used the same baseline variables as quoted in Mega et al. for multivariable adjustment.

### Genotype quality control

Variants were excluded if they had a call rate < 95%, deviated substantially from Hardy–Weinberg equilibrium (*p* < 10^−6^), [9] or had a MAF of less than 1%. After quality control procedures, a total of 338 variants were available for analysis in all 4932 individuals.

### Statistical analysis

Associations between SNPs and outcomes were assessed individually using adjusted proportional hazards regression models. The choice of potential covariates was based on our previous analyses which stratified risk for fatal as well as non-fatal outcomes [10]. SNPs that remained significant using a cut-off of *p* < 0.01 were reported. The Bonferroni method of adjusting for multiple comparisons suggested a cut-off of 0.0001. As no SNPs met the predetermined cut-off of *p* < 0.0001, the less conservative cut-off of 0.01 was used for retention of SNPs in the models.

The SNPs that were independent predictors for each cause of death in the LIPID data were used to create a risk score for each patient, applying the log of the hazard ratio from a model adjusted for clinical risk factors. The resulting scores were divided into quintiles and then the three middle groups were combined into one group. For the validation of the Mega et al. model, this procedure was repeated for CHD death using the SNPs and odds ratios previously reported in their manuscript [8].

### Ethics

The LIPID trial was approved by the ethics committee at each participating site. All patients gave written informed consent for cohort follow-up, either in the clinic or remotely. The LIPID Genetic cohort study was approved by the Human Research Ethics Committee of Sir Charles Gairdner Hospital, Perth. The Long term follow-up of patients in the LIPID cohort study was approved by University of Sydney Human Research Ethics Committee Reference No: 01-2002/2454. The Genetic Cohort study was approved by The Human Research Ethics Committee of Sir Charles Gairdner Hospital, Perth HREC No 2011-060.

### Patient involvement

Patients were not involved in the study design. Patient involvement in the study occurred at the time of informed consent, supervised by the Human Research Ethics Committee of each site.

## Results

### Characteristics of the study population

The baseline characteristics of the patients in the LIPID Genetic cohort (n = 4932) at the time of entry into the LIPID trial are summarised in Additional file 1: Table S2 and were similar to those of the full LIPID trial cohort [5–7].

### Outcomes during follow-up

Table 2 shows cause-specific deaths in the 10-year follow-up of the LIPID Genetic cohort. There was a total of 1558 deaths, of which 898 were cardiovascular, including 727 related to CHD, and 375 due to cancer.

**Table 2:** Risk stratification for cause-specific deaths over 10 years derived from the single nucleotide polymorphisms (SNPs) with the highest and lowest hazard ratios (HR) with an association stronger than the predetermined threshold of *p* < 0.01, unadjusted for baseline risks or multiple testing

Outcome variable	SNP number	HR* 95% CI	*p* Value	Genotype	Gene	Chromosome	Location	Function
Total deaths	rs2247056	1.15 (1.07, 1.25)	0.0003	CT	HLA-C/HLA-B	6	31,265,490	Blood lipid levels
	rs2131925	1.13 (1.05, 1.22)	0.0019	GT	DOCK7	1	63,025,942	Blood lipid levels
	rs10455872	1.19 (1.06, 1.34)	0.0038	AG	LPA	6	161,010,118	Blood Lp (a) level
	rs7298565	1.10 (1.03, 1.18)	0.0052	AG	UBE3B	12	109,937,534	DNA synthesis and cell proliferation
	rs7134594	0.91 (0.85, 0.97)	0.0060	CT	MMAB	12	110,000,193	Vitamin B12 metabolism
	rs16868846	0.82 (0.71, 0.95)	0.0068	CG	KCNK5	6	39,207,558	Potassium channel control
	rs2252641	1.10 (1.03, 1.18)	0.0080	AG	PABPCP2	2	145,801,461	CHD
CVD deaths	rs10455872	1.28 (1.10, 1.49)	0.0015	AG	LPA	6	161,010,118	Blood Lp (a) level; CHD
CHD deaths	rs10455872	1.33 (1.13, 1.57)	0.0008	AG	LPA	6	161,010,118	Lipoprotein (a) and LpPLA2 levels
Cancer deaths	rs2131925	1.28 (1.09, 1.50)	0.0022	GT	DOCK7	1	63,025,942	Blood lipid levels
	rs11556924	0.80 (0.68, 0.93)	0.0037	CT	ZC3HC1	7	129,663,496	CHD
	rs2247056	1.25 (1.07, 1.46)	0.0047	CT	HLA-C/HLA-B	6	31,265,490	Blood lipid levels

* Hazard ratios discovered from the data, not the odds ratios previously published in GWAS reports. Unadjusted for baseline risks or for multiple testing

### Association of individual SNPs with 10-year fatal outcomes in the LIPID Genetic cohort

**Fig. 1:**
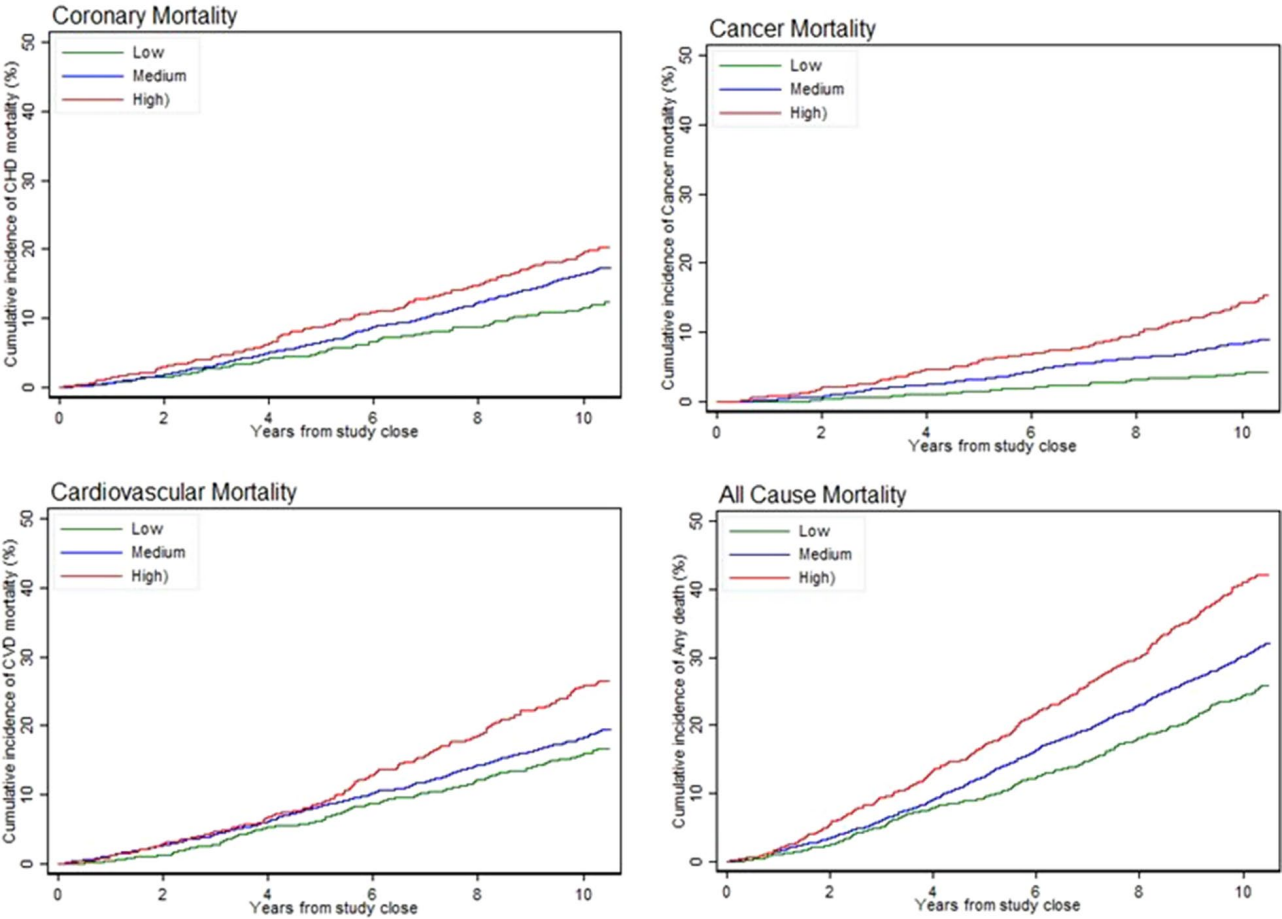
Plots of all-cause, coronary, cardiovascular, and cancer deaths over 10 years based on hazard ratios of risk for patients with high (top quintile), moderate (middle 3 quintiles) and low (lowest quintile) risk. Risk stratification derived from associations of SNPs with statistically significant hazard ratios with outcomes on unadjusted analyses

The associations (unadjusted for baseline variables or multiple testing) of fatal outcomes over 10 years from the end of the double-blind phase of the RCT with the individual SNPs are presented in Table 2. After adjustment for baseline variables, and after further correction for multiple SNP testing, there were no statistically significant associations of individual SNPs with subsequent deaths. When testing for internal validation, the risk score for each patient was based on the hazard ratios discovered from our own data. In this *internal validation*, we found highly significant stratification of risk, including for deaths from cancer, which had an adjusted hazard ratio of 3.8 (95% confidence interval 2.59–5.57, *p* = 7.82 × 10^−12^) between the lowest and highest quintile (Table 3). The pattern for each of the 10-year fatal outcomes examined is displayed graphically in Fig. 1.

**Table 3:** 10-year fatal outcomes by risk categories based on the ranking of hazard ratios discovered from the data

Variable	Model	Level of risk variable	No. Tot % deaths	HR (95% CI)	*p* Value	Overall *p* value
All-cause deaths	Unadjusted	Low	241/974 (25%)	1		4.995E−14
		Moderate	897/2918 (31%)	1.30 (1.12, 1.49)	0.0003503	
		High	391/973 (40%)	1.85 (1.57, 2.17)	7.438E−14	
	Adjusted^*^	Low		1		3.0937E−8
		Moderate		1.27 (1.10, 1.47)	0.0009637	
		High		1.62 (1.37, 1.90)	6.9682E−9	
Coronary deaths	Unadjusted	Low	108/999 (11%)	1		0.0000269
		Moderate	439/2905 (15%)	1.44 (1.16, 1.77)	0.0007483	
		High	175/975 (18%)	1.75 (1.38, 2.23)	4.5738E−6	
	Adjusted^*^	Low		1		0.0003085
		Moderate		1.37 (1.11, 1.70)	0.0032343	
		High		1.64 (1.29, 2.09)	0.0000588	
CVD deaths	Unadjusted	Low	151/1,015 (15%)	1		2.0018E−6
		Moderate	504/2869 (18%)	1.19 (1.00, 1.43)	0.0555253	
		High	225/968 (23%)	1.66 (1.35, 2.03)	1.6724E−6	
	Adjusted^*^	Low		1		0.0002765
		Moderate		1.16 (0.96, 1.39)	0.1162958	
		High		1.50 (1.22, 1.85)	0.0001406	
Cancer deaths	Unadjusted	Low	35/977 (4%)	1		4.17E−12
		Moderate	219/2928 (7%)	2.18 (1.53, 3.12)	0.0000179	
		High	120/976 (12%)	3.74 (2.57, 5.45)	6.559E−12	
	Adjusted^*^	Low		1		4.429E−12
		Moderate		2.20 (1.53, 3.17)	0.0000191	
		High		3.80 (2.59, 5.57)	7.819E−12	

Note that these are not the odds ratios previously published in GWAS reports. The hazard ratios (mean and 95% CI) for the moderate and high-risk categories are compared with the low-risk category

Hazard ratios are shown unadjusted and adjusted for baseline risks (*): Rx with pravastatin, age, aspirin, atrial fibrillation, total cholesterol, high-density lipoprotein cholesterol, dyspnoea, angina, peripheral vascular disease, diabetes mellitus, hypertension, history of myocardial infarction, obesity, history of coronary revascularisation, sex, current smoking, stroke, aspirin, systolic blood pressure, diastolic blood pressure, fasting glucose and white blood count. High-risk = top quintile, moderate risk = middle three quintiles, low risk = bottom quintile

*HR* hazard ratio, *CI* confidence interval

### Prediction of 10-year risk of fatal CHD from a previously-derived GRS

**Fig. 2:**
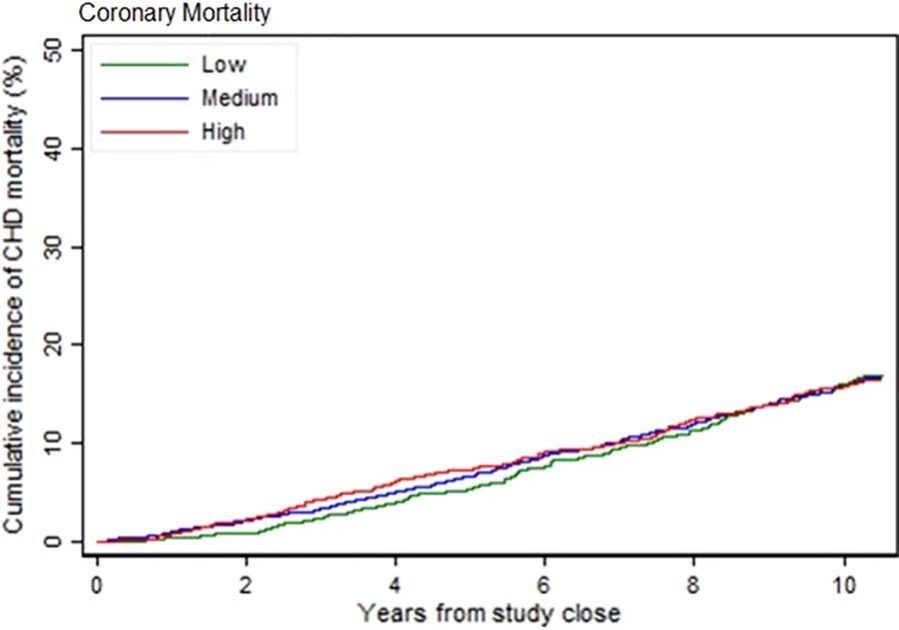
Plot of coronary heart disease death over 10 years using the genetic risk score derived by Mega et al. [8]. High risk = top quintile, moderate risk = middle three quintiles, low risk = bottom quintile. Unadjusted for baseline variables

The GRS described by Mega et al. [8] both unadjusted and after adjustment for the baseline risk factors listed in that publication, showed no statistically significant stratification for CHD death over 10 years. The results for CHD death are shown in Fig. 2. Before and after adjustment for baseline variables, the categories of risk based on each individual’s GRS did not distinguish between high (top quintile) moderate (middle 3 quintiles) and low (bottom quintile) risk of CHD death over 10 years (Table 4). The variables adjusted for are described in the Table.

**Table 4:** Levels of risk of CHD deaths over 10 years and recurrent CHD events over 2 years, when applying the Mega et al. ^¥^ GRS to the LIPID Genetic cohort

Variable	Level of risk	No/total %events	Unadjusted for risk factors			Adjusted for risk factors as in Mega et al.^¥¥^		
			HR (95% CI)	*p* Value	Overall *p* value	HR (95% CI)	*p* Value	Overall *p* value
CHD death over 10 years	Low	142/976 (15%)	1		0.99	1		0.59
	Moderate	429/2928 (15%)	1.01 (0.84, 1.22)	0.92		1.02 (0.85, 1.24)	0.81	
	High	144/976 (15%)	1.01 (0.80, 1.28)	0.90		1.12 (0.86, 1.41)	0.35	
CHD events ^¥^ as described in Mega et al. (10) over 2 years	Low	102/976 (10%)	1		0.39	1		0.29
	Moderate	328/2928 (11%)	1.13 (0.91, 1.41)	0.27		1.14 (0.92, 1.43)	0.23	
	High	116/976 (12%)	1.20 (0.92, 1.57)	0.18		1.23 (0.94, 1.61)	0.12	

Low, moderate or high levels of risk were determined by the risk score for each patient, calculated from the previously published 27 SNP GRS of Mega et al. (8) The hazard ratios (mean with 95% CI) compare the low risk (lowest quintile) with moderate risk (middle 3 quintiles) and high-risk (top quintile) categories

The results are shown unadjusted and then adjusted for the same baseline variables used in calculation of the Mega et al. [8] GRS

(^¥¥^ history of hypertension, diabetes mellitus, sex, age, current smoking, low-density lipoprotein cholesterol, high-density lipoprotein cholesterol.) High-risk = top quintile, moderate risk = middle 3 quintiles, low risk = bottom quintile. ^¥^CHD events as defined by Mega et al. were CHD death, non-fatal MI, unstable angina pectoris, coronary artery bypass graft and percutaneous coronary intervention). *CI* confidence interval, *HR* hazard ratio

### Two-year non-fatal outcomes

We also examined the value of the same SNPs for predicting non-fatal as well as fatal outcomes in the two years of open label follow-up, using a composite of CHD events (CHD death, non-fatal myocardial infraction, unstable angina, coronary artery bypass grafting and percutaneous coronary revascularisation) as the principal outcome measure, as described by Mega et al. [8] (Table 5).

**Table 5:** Testing the GRS published by Mega et al. [8] using the same 27 SNPs to predict the same outcomes (CHD death, non-fatal MI, UAP, CABG and PCI) at 2 years

Model	27 SNP GRS of Mega et al. added to the model		
	NRI	Baseline C-statistic	C-statistic + SNPs
Base model: hypertension, low-density lipoprotein cholesterol, high-density lipoprotein cholesterol, diabetes, sex, age, current smoking	0.097	0.69	0.70
Base model as above with history of CHD included	0.064	0.67	0.69
Base model as above with history of prior MI included (0 = none, 1 = one, 2 = multiple)	0.067	0.69	0.70

Effect on NRI and C-statistic determined after adding the previously published GRS to three different models

*CHD* coronary heart disease, *MI* myocardial infarction

### Reclassification

When the 27 SNPs were added to a model with the baseline risk factors used by Mega et al. [8] there was moderate improvement in the net reclassification index (NRI) with a value of 0.097 and a very minor increase in the C-statistic from the ROC (receiver operating curve) curve from 0.69 to 0.70. When we added a history of prior CHD or prior MI (none, one or multiple) to the baseline model, with and without the SNPs, there were NRIs of 0.064 and 0.067, respectively (Table 5), and there was no significant change in the C-statistic.

## Discussion

Our results do not support the hypothesis that individual SNPs strongly associated with prevalent or incident CHD on GWAS, or a previously validated 27-SNP GRS based on these SNPs [8] can predict long-term outcomes of patients with CHD who have had an ACS in the past.

### The role of individual SNPs

In the early GWAS reports of associations of SNPs with CHD, *p* values < 10^−10^ were found for multiple SNPs, particularly the rs1333049 variant in the 9p21 gene [11]. We have significantly extended these previous observations by selecting a large number of other SNPs that have shown statistically strong associations with CHD or CHD pathways in previous GWAS reports. Our conclusion from this first part of our study is that selecting individual SNPs from strong associations with CHD in GWAS did not improve prediction of long-term cardiovascular risk in established CHD.

### The role of a previously validated GRS based on 27 SNPs

We next tested a previously validated GRS for its potential for clinical application to improve risk prediction in known CHD patients [8]. Since the risk score for each patient was derived from the hazard ratios within the data set, it was expected that ranking of the risk scores would correlate strongly with cardiovascular risk, and this was indeed the case, even after adjustment for clinical variables. The strong correlation with cancer deaths was unexpected. However, a more stringent test of the role of a genetic influence on outcome is to test if an externally derived GRS is predictive.

GRS ranging from 19 to 300 SNPs [12] and more recently, PRS (Polygenic Risk Scores) of 50,000 [13] to 6 million SNPs [14] have been evaluated for their value in identifying risk of incident CHD. The larger panels have been shown to be superior to smaller scale scores in predicting events in people at high risk of incident CHD, but recent reports show only a modest improvement in prediction over clinical predictors [15].

GRSs developed for prediction of recurrent events in known CHD patients have been tested in smaller cohorts than the present study, less well characterised to enable full adjustment for confounding, or with shorter follow-up, and external validation has been infrequent [16]. Modest associations with recurrent events have been shown, but none have demonstrated clear-cut improvement in risk prediction in patients with established vascular disease [1, 16–23]. These are summarised in Table 6.

**Table 6:** Summary of LIPID genetic study and previous studies assessing GRS in prediction of secondary cardiovascular risk in patients with documented cardiovascular disease and post-ACS

Author	References	Patients	n	Duration of follow-up (years)	CV events	Fatal CV events	Type of GRS	Independent association with CV events
Patel et al. 2012	[16]	CAD on angiography	2597	2.5	358	257	11 SNPs	No independent association
Weijmans et al. 2015	[17]	Symptomatic vascular disease	5742	6.5	933	N/R	30 SNPs	No independent association
Labos et al. 2015	[18]	ACS	3503	1	389	N/R	30 SNPs	No independent association
Vaara et al. 2015	[19]	ACS with coronary angiography	2090	5.5	263	N/R	47 SNPs 153 SNPs	GRS 47. Adj HR 1.17; 95% CI, 1.01–1.36 *p* = 0.037. No improvement in C-statistic GRS 153. No association
Mega et al. 2015	[8]	Recent ACS (CARE)	2878	4.94	330	N/R	27 SNPs	HR 1.14 (0.98–1.32) per SD of GRS *p* = 0.081
Mega et al. 2015	[8]	Recent ACS (PROVE-IT)	1999	2.03	22	N/R	27 SNPs	HR 1.14 (0.95–1.36) per SD of GRS *p* = 0.15
Christiansen et al. 2017	[20]	High-risk stable CHD	879	2.8	N/R	N/R	45 SNPs	Adj. HR 1.50 (95% CI 1.00–2.25) for all CV events including revascularisation. Risks of CV death and all-cause death unaffected
Wirtwein et al. 2017	[21]	CHD on angiography	1345	8.6	882	114	19 SNPs	Inconsistent
Pereira et al. 2017	[22]	CHD Southern Europe	1464	4.9	N/R	107	32 SNPs	No independent association
Jiang et al. 2020	[23]	Chinese patients Recent ACS	1667	2	N/R	N/R	79 SNPs	HR 1.33 (1.10–1.61) P = 0.003 per SD of GRS; slight increase in discrimination after adjustment for clinical factors
Current study		CHD, distant ACS	4932	> 10	554 (2 years)	898 (10 years)	27 SNPs (same as Mega et al.)	No independent association No improvement in NRI or C-statistic

*ACS* acute coronary syndromes, *AMI* acute myocardial infarction, *CAD* coronary artery disease, *CHD* coronary heart disease, *CVD* cardiovascular disease, *CARE* Cholesterol and Coronary Events trial, *PROVE-IT* Pravastatin or Atorvastatin Evaluation and Infection Therapy, *HR* hazard ratio, *GRS* genetic risk score, *CI* confidence interval, *Adj* adjusted, *N/R* not reported

Because of the inherent appeal of a clinically applicable GRS with a limited number of SNPs we chose to evaluate the 27 SNP GRS which had been derived by Mega et al. [8] and which has been externally validated for predicting incident CHD [24]. This GRS was derived from large number of patients with established CHD including nearly 5000 who were in the CARE [25] and PROVE-IT [26] clinical trials of statin therapy. When the Mega et al. [8] score was applied to the LIPID cohort, it did not show any genetic contribution to prediction of recurrent CHD events or of fatal outcomes even before adjustment for clinical determinants of risk. We conclude from this second part of our study that there is a low likelihood of identifying CHD patients at high risk of recurrent events based on GRSs composed of an abbreviated SNP panel.

### Limitations

There are several limitations of this study. The analyses are subject to recruitment bias which would be relevant if we were studying early survival after ACS but is less relevant for a longer-term study of a defined CHD cohort whose ACS was years distant.

We chose only one previously described GRS for validation. The GRS derived by Mega et al. [8] was the most relevant score for testing against outcomes in our cohort as it included patients with a similar clinical profile although the endpoints in the Mega GRS were only for the duration of the clinical trials. The Mega et al. database included primary prediction studies but also included 17,000 person years of follow-up in secondary prediction studies.

The reasons why this study of genetic polymorphisms of individual genes did not reveal an effect on the risk of recurrent events in patients with CAD remains unclear. Firstly, the sample size in this study may have been too small to detect a genetic effect on outcomes, but with a total of over 1500 deaths, this seems unlikely. Secondly, many of the clinical variables used for adjustment in the statistical models, are themselves subject to genetic influence, but it is striking that the lack of prediction by genetic variants was observed even before adjustment for clinical predictors. Finally, CHD, particularly when an ACS has occurred in the past, may simply be too complex a condition for genetics to influence survival.

It is important to recognise that our data do not exclude a genetic influence on survival in CHD patients. However, it is clear from these analyses that clinically applicable genetic profiling with single SNPs or a SNP-derived GRS with a limited number of highly selected SNPs did not add precision to the prediction of recurrent major CHD outcomes. It is conceivable that a polygenic risk score (PRS) with many thousands of SNPs will demonstrate a genetic influence on outcomes, but it remains to be established whether a PRS will have a clinically applicable role in enhancing the precision of recurrent event prediction beyond clinical markers of risk [27]. Further studies to clarify the genetic contribution to risk in established CHD will require the pooling of data from large numbers of individual cohorts [28], recognition of the limitations of GWAS [29] and possibly an omnigenic approach with exploration of regulatory genes undetected on GWAS [30].

## Conclusion

In this large cohort of patients with CHD who had an ACS in the past, individual SNPs strongly associated with prevalent or incident CHD on GWAS, and a previously validated 27-SNP GRS based on these SNPs did not predict long-term outcomes.

## Supplementary Information

**Additional file 1:** SNPs included in the custom designed Illumina Gold Gate array of 384 SNPs with minor allele frequency (MAF) > 1 plus additional SNPs included in the panel derived by Mega et al [8].

## Data Availability

The datasets used and/or analysed during the current study are available from the corresponding author on reasonable request by emailing peter.thompson@health.wa.gov.au.

## Abbreviations

SNP: Single nucleotide polymorphism
GWAS: Genome wide association study
CHD: Coronary heart disease
ACS: Acute coronary syndrome
MI: Myocardial infarction.
GRS: Genetic risk score
PRS: Polygenic risk score
LIPID trial: Long-Term Intervention with Pravastatin in Ischaemic Disease trial
NRI: Net reclassification index
NHMRC: National Health and Medical Research Council of Australia
ROC: Receiver operating curve
