# Supplementary material for: Common genetic variants do not predict recurrent events in coronary heart disease patients
Source: BMC Cardiovasc Disord. 2022 Mar 9;22:96. doi: 10.1186/s12872-022-02520-0 (PMC8908687; doi:10.1186/s12872-022-02520-0)
Supplement: Supplementary file 1 — Additional file 1: SNPs included in the custom designed Illumina Gold Gate array of 384 SNPs with minor allele frequency (MAF) > 1 plus additional SNPs included in the panel derived by Mega et al [8]. [file 12872_2022_2520_MOESM1_ESM.docx]

**SUPPLEMENTARY MATERIAL**

**Supplementary Table 1** SNPs included in the custom designed Illumina GoldGate array of 384 SNPs with minor allele frequency (MAF) >1 plus 5 additional SNPs included in the panel derived by Mega et al. [8]

| Locus_Name | Genome_Build_Version | Chromosome | Coordinate | Sequence_Orientation | Gene_Symbol | Location |
| --- | --- | --- | --- | --- | --- | --- |
| rs225132 | 37.1 | 1 | 8095500 | FORWARD | SLC45A1/ERRFI1 | INTERGENIC |
| rs12027135 | 37.1 | 1 | 25775733 | FORWARD | TMEM57 | INTRON |
| rs4660293 | 37.1 | 1 | 40028180 | FORWARD | PABPC4 | INTRON |
| rs12037222 | 37.1 | 1 | 40064961 | FORWARD | HEYL/PABPC4 | INTERGENIC |
| rs11206510 | 37.1 | 1 | 55496039 | FORWARD | BSND/PCSK9 | INTERGENIC |
| rs2479409 | 37.1 | 1 | 55504650 | FORWARD | BSND/PCSK9 | INTERGENIC |
| rs11591147 | 37.1 | 1 | 55505647 | FORWARD | PCSK9 | CODING |
| rs17114036 | 37.1 | 1 | 56962821 | FORWARD | PPAP2B | INTRON |
| rs17114046 | 37.1 | 1 | 56966350 | FORWARD | PPAP2B | INTRON |
| rs2131925 | 37.1 | 1 | 63025942 | FORWARD | DOCK7 | INTRON |
| rs3850634 | 37.1 | 1 | 63050598 | FORWARD | DOCK7 | INTRON |
| rs4420065 | 37.1 | 1 | 66161461 | FORWARD | LEPR/PDE4B | INTERGENIC |
| rs2916 | 37.1 | 1 | 67216697 | REVERSE | SGIP1/TCTEX1D1 | INTERGENIC |
| rs660240 | 37.1 | 1 | 109817838 | REVERSE | CELSR2 | UTR |
| rs629301 | 37.1 | 1 | 109818306 | REVERSE | CELSR2 | UTR |
| rs646776 | 37.1 | 1 | 109818530 | REVERSE | PSRC1/CELSR2 | INTERGENIC |
| rs599839 | 37.1 | 1 | 109822166 | FORWARD | CELSR2/PSRC1 | INTERGENIC |
| rs11240065 | 37.1 | 1 | 146940196 | FORWARD | BCL9/CHD1L | INTERGENIC |
| rs4845625 | 37.1 | 1 | 154422067 | FORWARD | IL6R | INTRON |
| rs4129267 | 37.1 | 1 | 154426264 | FORWARD | IL6R | INTRON |
| rs2794520 | 37.1 | 1 | 159678816 | FORWARD | CRP/APCS | INTERGENIC |
| rs1801274 | 37.1 | 1 | 161479745 | REVERSE | FCGR2A | COMPLEX |
| rs12143842 | 37.1 | 1 | 162033890 | FORWARD | NOS1AP/OLFML2B | INTERGENIC |
| rs1689800 | 37.1 | 1 | 182168885 | REVERSE | LOC100130996 | INTRON |
| rs4335430 | 37.1 | 1 | 217100231 | FORWARD | ESRRG | INTRON |
| rs2807834 | 37.1 | 1 | 220970593 | FORWARD | MOSC1 | INTRON |
| rs11118620 | 37.1 | 1 | 221028508 | FORWARD | LOC100129376/HLX | INTERGENIC |
| rs17464857 | 37.1 | 1 | 222762709 | FORWARD | TAF1A | INTRON |
| rs17465637 | 37.1 | 1 | 222823529 | FORWARD | MIA3 | INTRON |
| rs4846914 | 37.1 | 1 | 230295691 | FORWARD | GALNT2 | INTRON |
| rs1321257 | 37.1 | 1 | 230305312 | FORWARD | GALNT2 | INTRON |
| rs514230 | 37.1 | 1 | 234858597 | REVERSE | PP2672/IRF2BP2 | INTERGENIC |
| rs12239046 | 37.1 | 1 | 247601595 | FORWARD | NLRP3 | INTRON |
| rs6548238 | 37.1 | 2 | 634905 | FORWARD | LOC727944/TMEM18 | INTERGENIC |
| rs7371392 | 37.1 | 2 | 3083625 | REVERSE | MYT1L/LOC729897 | INTERGENIC |
| rs10169682 | 37.1 | 2 | 3086833 | FORWARD | MYT1L/LOC729897 | INTERGENIC |
| rs12714371 | 37.1 | 2 | 3087454 | FORWARD | LOC729897/MYT1L | INTERGENIC |
| rs4854189 | 37.1 | 2 | 3098843 | REVERSE | LOC729897/MYT1L | INTERGENIC |
| rs1453822 | 37.1 | 2 | 3099075 | FORWARD | LOC729897/MYT1L | INTERGENIC |
| rs515135 | 37.1 | 2 | 21286057 | FORWARD | LOC100129278/APOB | INTERGENIC |
| rs1260326 | 37.1 | 2 | 27730940 | FORWARD | GCKR | CODING |
| rs4299376 | 37.1 | 2 | 44072576 | FORWARD | ABCG8 | INTRON |
| rs6544713 | 37.1 | 2 | 44073881 | FORWARD | ABCG8 | INTRON |
| rs13407662 | 37.1 | 2 | 53782559 | FORWARD | LOC730100/ASB3 | INTERGENIC |
| rs1561198 | 37.1 | 2 | 85809989 | REVERSE | VAMP8/VAMP5 | INTERGENIC |
| rs6734238 | 37.1 | 2 | 113841030 | FORWARD | IL1F10/IL1RN | INTERGENIC |
| rs12464355 | 37.1 | 2 | 118849850 | FORWARD | INSIG2 | INTRON |
| rs13418717 | 37.1 | 2 | 127662897 | FORWARD | LOC339760/BIN1 | INTERGENIC |
| rs7570971 | 37.1 | 2 | 135837906 | FORWARD | RAB3GAP1 | INTRON |
| rs6759321 | 37.1 | 2 | 136322676 | FORWARD | R3HDM1 | INTRON |
| rs2252641 | 37.1 | 2 | 145801461 | REVERSE | LOC100131409/PABPCP2 | INTERGENIC |
| rs174230 | 37.1 | 2 | 160175310 | REVERSE | BAZ2B/WDSUB1 | INTERGENIC |
| rs4665058 | 37.1 | 2 | 160190209 | FORWARD | BAZ2B | INTRON |
| rs12328675 | 37.1 | 2 | 165540800 | FORWARD | COBLL1/GRB14 | INTERGENIC |
| rs6725887 | 37.1 | 2 | 203745885 | FORWARD | WDR12 | INTRON |
| rs2943645 | 37.1 | 2 | 227099180 | FORWARD | KIAA1486/IRS1 | INTERGENIC |
| rs2972146 | 37.1 | 2 | 227100698 | REVERSE | IRS1/KIAA1486 | INTERGENIC |
| rs1515100 | 37.1 | 2 | 227128917 | FORWARD | IRS1/KIAA1486 | INTERGENIC |
| rs11563251 | 37.1 | 2 | 234679384 | FORWARD | UGT1A1/UGT1A4/UGT1A9/UGT1A10/UGT1A6/UGT1A8/UGT1A7/UGT1A5/UGT1A3 | COMPLEX |
| rs12631819 | 37.1 | 3 | 12342861 | FORWARD | PPARG | INTRON |
| rs2290159 | 37.1 | 3 | 12628920 | FORWARD | RAF1 | INTRON |
| rs6763538 | 37.1 | 3 | 16323236 | FORWARD | OXNAD1 | INTRON |
| rs7432308 | 37.1 | 3 | 18877562 | FORWARD | KCNH8/LOC100132345 | INTERGENIC |
| rs4687718 | 37.1 | 3 | 53282303 | FORWARD | TKT | INTRON |
| rs7431862 | 37.1 | 3 | 65250132 | FORWARD | MAGI1/ADAMTS9 | INTERGENIC |
| rs645040 | 37.1 | 3 | 135926622 | FORWARD | PCCB/MSL2 | INTERGENIC |
| rs13324341 | 37.1 | 3 | 138070901 | FORWARD | MRAS | INTRON |
| rs2306374 | 37.1 | 3 | 138119952 | FORWARD | MRAS | INTRON |
| rs9818870 | 37.1 | 3 | 138122122 | FORWARD | MRAS | UTR |
| rs16851055 | 37.1 | 3 | 140799213 | FORWARD | SPSB4 | INTRON |
| rs1523288 | 37.1 | 3 | 165280209 | REVERSE | SLITRK3/BCHE | INTERGENIC |
| rs35898760 | 37.1 | 3 | 171950181 | FORWARD | FNDC3B | INTRON |
| rs6445051 | 37.1 | 3 | 171954098 | FORWARD | FNDC3B | INTRON |
| rs35198260 | 37.1 | 3 | 187718139 | FORWARD | LOC339929/BCL6 | INTERGENIC |
| rs17621680 | 37.1 | 4 | 24749535 | FORWARD | DHX15/SOD3 | INTERGENIC |
| rs12505599 | 37.1 | 4 | 24750934 | FORWARD | DHX15/SOD3 | INTERGENIC |
| rs6844183 | 37.1 | 4 | 28821006 | FORWARD | PCDH7/STIM2 | INTERGENIC |
| rs442177 | 37.1 | 4 | 88030261 | REVERSE | AFF1 | INTRON |
| rs13107325 | 37.1 | 4 | 103188709 | FORWARD | SLC39A8 | COMPLEX |
| rs2200733 | 37.1 | 4 | 111710169 | FORWARD | LOC729065/PITX2 | INTERGENIC |
| rs1906599 | 37.1 | 4 | 111712686 | FORWARD | PITX2/LOC729065 | INTERGENIC |
| rs1878406 | 37.1 | 4 | 148393664 | FORWARD | EDNRA/LOC100287219 | INTERGENIC |
| rs6834179 | 37.1 | 4 | 155440450 | FORWARD | DCHS2/PLRG1 | INTERGENIC |
| rs4482740 | 37.1 | 4 | 155446305 | FORWARD | PLRG1/DCHS2 | INTERGENIC |
| rs7692387 | 37.1 | 4 | 156635309 | FORWARD | GUCY1A3 | INTRON |
| rs11748327 | 37.1 | 5 | 4029789 | FORWARD | LOC340094/IRX1 | INTERGENIC |
| rs2967951 | 37.1 | 5 | 10464107 | REVERSE | ROPN1L | INTRON |
| rs6450176 | 37.1 | 5 | 53298025 | FORWARD | ARL15 | INTRON |
| rs9686661 | 37.1 | 5 | 55861786 | FORWARD | MAP3K1/LOC100289077 | INTERGENIC |
| rs12916 | 37.1 | 5 | 74656539 | FORWARD | HMGCR | UTR |
| rs273909 | 37.1 | 5 | 131667353 | REVERSE | SLC22A4 | INTRON |
| rs2706399 | 37.1 | 5 | 131867702 | FORWARD | IRF1/IL5 | INTERGENIC |
| rs10059999 | 37.1 | 5 | 144393385 | FORWARD | PRELID2/KCTD16 | INTERGENIC |
| rs11746929 | 37.1 | 5 | 149133874 | FORWARD | PPARGC1B | INTRON |
| rs6882076 | 37.1 | 5 | 156390297 | FORWARD | HAVCR1/TIMD4 | INTERGENIC |
| rs1553318 | 37.1 | 5 | 156479323 | FORWARD | HAVCR1 | INTRON |
| rs17507623 | 37.1 | 6 | 11401519 | FORWARD | TMEM170B/NEDD9 | INTERGENIC |
| rs2235388 | 37.1 | 6 | 11773710 | REVERSE | C6orf105 | INTRON |
| rs4714955 | 37.1 | 6 | 12903435 | FORWARD | PHACTR1 | INTRON |
| rs9349379 | 37.1 | 6 | 12903957 | FORWARD | PHACTR1 | INTRON |
| rs1014342 | 37.1 | 6 | 12923157 | FORWARD | PHACTR1 | INTRON |
| rs12526453 | 37.1 | 6 | 12927544 | FORWARD | PHACTR1 | INTRON |
| rs3757354 | 37.1 | 6 | 16127407 | REVERSE | DTNBP1/MYLIP | INTERGENIC |
| rs6924995 | 37.1 | 6 | 16161425 | FORWARD | GMPR/MYLIP | INTERGENIC |
| rs2142672 | 37.1 | 6 | 16197194 | REVERSE | MYLIP/GMPR | INTERGENIC |
| rs497733 | 37.1 | 6 | 23672259 | REVERSE | LOC389370/LOC100129616 | INTERGENIC |
| rs1800562 | 37.1 | 6 | 26093141 | FORWARD | HFE | COMPLEX |
| rs6929846 | 37.1 | 6 | 26458265 | FORWARD | BTN2A1 | UTR |
| rs3869109 | 37.1 | 6 | 31184196 | REVERSE | HCG27/HLA-C | INTERGENIC |
| rs2247056 | 37.1 | 6 | 31265490 | FORWARD | HLA-C/HLA-B | INTERGENIC |
| rs389883 | 37.1 | 6 | 31947460 | REVERSE | STK19 | INTRON |
| rs3177928 | 37.1 | 6 | 32412435 | FORWARD | HLA-DRA | UTR |
| rs2814982 | 37.1 | 6 | 34546560 | FORWARD | SPDEF/C6orf106 | INTERGENIC |
| rs2814944 | 37.1 | 6 | 34552797 | FORWARD | SPDEF/C6orf106 | INTERGENIC |
| rs17609940 | 37.1 | 6 | 35034800 | FORWARD | ANKS1A | INTRON |
| rs10947789 | 37.1 | 6 | 39174922 | FORWARD | KCNK5 | INTRON |
| rs16868846 | 37.1 | 6 | 39207558 | FORWARD | KCNK5/KCNK17 | INTERGENIC |
| rs2815063 | 37.1 | 6 | 39262535 | FORWARD | KCNK5/KCNK17 | INTERGENIC |
| rs556621 | 37.1 | 6 | 44594159 | REVERSE | LOC100128935/SUPT3H | INTERGENIC |
| rs527461 | 37.1 | 6 | 49725341 | FORWARD | CRISP3/PGK2 | INTERGENIC |
| rs489184 | 37.1 | 6 | 49747462 | REVERSE | CRISP3/PGK2 | INTERGENIC |
| rs11153594 | 37.1 | 6 | 116354591 | FORWARD | LOC728402/FRK | INTERGENIC |
| rs11153730 | 37.1 | 6 | 118667522 | FORWARD | LOC644303/SLC35F1 | INTERGENIC |
| rs11970286 | 37.1 | 6 | 118680374 | FORWARD | LOC644303/SLC35F1 | INTERGENIC |
| rs7769153 | 37.1 | 6 | 131256364 | FORWARD | EPB41L2 | INTRON |
| rs2327429 | 37.1 | 6 | 134209837 | FORWARD | TCF21/MGC34034 | INTERGENIC |
| rs12190287 | 37.1 | 6 | 134214525 | FORWARD | LOC100288120/TCF21 | COMPLEX |
| rs12204317 | 37.1 | 6 | 134621215 | FORWARD | SGK1 | INTRON |
| rs9493891 | 37.1 | 6 | 134623840 | FORWARD | SGK1 | INTRON |
| rs605066 | 37.1 | 6 | 139829666 | FORWARD | CITED2/LOC729076 | INTERGENIC |
| rs2351542 | 37.1 | 6 | 155310743 | FORWARD | TIAM2/RBM16 | INTERGENIC |
| rs1564348 | 37.1 | 6 | 160578860 | FORWARD | SLC22A1 | INTRON |
| rs3106172 | 37.1 | 6 | 160727975 | FORWARD | SLC22A2/SLC22A3 | INTERGENIC |
| rs2048327 | 37.1 | 6 | 160863532 | FORWARD | SLC22A3 | INTRON |
| rs9355288 | 37.1 | 6 | 160867159 | FORWARD | SLC22A3 | INTRON |
| rs3123629 | 37.1 | 6 | 160906086 | FORWARD | LPAL2 | INTRON |
| rs3798220 | 37.1 | 6 | 160961137 | FORWARD | LPA | CODING |
| rs10455872 | 37.1 | 6 | 161010118 | REVERSE | LPA | INTRON |
| rs1084651 | 37.1 | 6 | 161089817 | FORWARD | PLG/LPA | INTERGENIC |
| rs4252120 | 37.1 | 6 | 161143608 | FORWARD | PLG | INTRON |
| rs11984041 | 37.1 | 7 | 19031935 | FORWARD | HDAC9 | INTRON |
| rs2023938 | 37.1 | 7 | 19036775 | FORWARD | HDAC9 | UTR |
| rs2107595 | 37.1 | 7 | 19049388 | REVERSE | HDAC9/TWIST1 | INTERGENIC |
| rs12670798 | 37.1 | 7 | 21607352 | FORWARD | DNAH11 | INTRON |
| rs13225783 | 37.1 | 7 | 27330056 | FORWARD | HIBADH/LOC100129463 | INTERGENIC |
| rs39075 | 37.1 | 7 | 29276692 | FORWARD | CHN2 | INTRON |
| rs2070971 | 37.1 | 7 | 44197583 | REVERSE | GCK | INTRON |
| rs2072183 | 37.1 | 7 | 44579180 | REVERSE | NPC1L1 | CODING |
| rs217386 | 37.1 | 7 | 44600695 | FORWARD | DDX56/NPC1L1 | INTERGENIC |
| rs13438050 | 37.1 | 7 | 48718056 | FORWARD | ABCA13/CDC14C | INTERGENIC |
| rs10258912 | 37.1 | 7 | 48718406 | FORWARD | CDC14C/ABCA13 | INTERGENIC |
| rs7811265 | 37.1 | 7 | 72934510 | FORWARD | BAZ1B | INTRON |
| rs13233571 | 37.1 | 7 | 72971231 | FORWARD | BCL7B | INTRON |
| rs17145738 | 37.1 | 7 | 72982874 | FORWARD | BCL7B/TBL2 | INTERGENIC |
| rs1859023 | 37.1 | 7 | 90240585 | REVERSE | PFTK1/CLDN12 | INTERGENIC |
| rs43061 | 37.1 | 7 | 95102534 | FORWARD | PON2/ASB4 | INTERGENIC |
| rs10953541 | 37.1 | 7 | 107244545 | FORWARD | BCAP29 | INTRON |
| rs11556924 | 37.1 | 7 | 129663496 | FORWARD | ZC3HC1 | CODING |
| rs4731702 | 37.1 | 7 | 130433384 | FORWARD | KLF14/FLJ43663 | INTERGENIC |
| rs12703165 | 37.1 | 7 | 151566756 | FORWARD | PRKAG2 | INTRON |
| rs4875812 | 37.1 | 8 | 1759466 | FORWARD | CLN8/ARHGEF10 | INTERGENIC |
| rs9987289 | 37.1 | 8 | 9183358 | FORWARD | PPP1R3B/TNKS | INTERGENIC |
| rs2126259 | 37.1 | 8 | 9185146 | FORWARD | TNKS/PPP1R3B | INTERGENIC |
| rs11776767 | 37.1 | 8 | 10683929 | FORWARD | PINX1 | INTRON |
| rs6983129 | 37.1 | 8 | 11591136 | FORWARD | GATA4 | INTRON |
| rs1961456 | 37.1 | 8 | 18255709 | FORWARD | NAT2 | INTRON |
| rs1495741 | 37.1 | 8 | 18272881 | FORWARD | NAT2/PSD3 | INTERGENIC |
| rs1495743 | 37.1 | 8 | 18273300 | FORWARD | NAT2/PSD3 | INTERGENIC |
| rs264 | 37.1 | 8 | 19813180 | FORWARD | LPL | INTRON |
| rs12678919 | 37.1 | 8 | 19844222 | FORWARD | SLC18A1/LPL | INTERGENIC |
| rs1030431 | 37.1 | 8 | 59311697 | FORWARD | FAM110B/UBXN2B | INTERGENIC |
| rs2081687 | 37.1 | 8 | 59388565 | REVERSE | CYP7A1/UBXN2B | INTERGENIC |
| rs6473383 | 37.1 | 8 | 83669120 | FORWARD | SNX16/RALYL | INTERGENIC |
| rs2293889 | 37.1 | 8 | 116599199 | FORWARD | TRPS1 | INTRON |
| rs2737229 | 37.1 | 8 | 116648565 | FORWARD | TRPS1 | INTRON |
| rs2954021 | 37.1 | 8 | 126482077 | FORWARD | TRIB1/LOC650095 | INTERGENIC |
| rs2954022 | 37.1 | 8 | 126482621 | FORWARD | TRIB1/LOC650095 | INTERGENIC |
| rs17321515 | 37.1 | 8 | 126486409 | FORWARD | TRIB1/LOC650095 | INTERGENIC |
| rs2954029 | 37.1 | 8 | 126490972 | FORWARD | TRIB1/LOC650095 | INTERGENIC |
| rs10808546 | 37.1 | 8 | 126495818 | FORWARD | TRIB1/LOC650095 | INTERGENIC |
| rs28540916 | 37.1 | 8 | 133959399 | FORWARD | TG | INTRON |
| rs4736614 | 37.1 | 8 | 133961011 | FORWARD | TG | INTRON |
| rs7015805 | 37.1 | 8 | 133965936 | FORWARD | TG | INTRON |
| rs9643296 | 37.1 | 8 | 133966348 | FORWARD | TG | INTRON |
| rs6988193 | 37.1 | 8 | 133980451 | FORWARD | TG | INTRON |
| rs7388248 | 37.1 | 8 | 144305353 | FORWARD | GPIHBP1/ZFP41 | INTERGENIC |
| rs11136341 | 37.1 | 8 | 145043543 | FORWARD | PLEC1 | INTRON |
| rs7024888 | 37.1 | 9 | 2636992 | FORWARD | VLDLR | INTRON |
| rs643531 | 37.1 | 9 | 15296034 | FORWARD | TTC39B | INTRON |
| rs581080 | 37.1 | 9 | 15305378 | FORWARD | TTC39B | INTRON |
| rs2210327 | 37.1 | 9 | 18109235 | FORWARD | ADAMTSL1/SH3GL2 | INTERGENIC |
| rs3217992 | 37.1 | 9 | 22003223 | REVERSE | CDKN2B | UTR |
| rs7865618 | 37.1 | 9 | 22031005 | FORWARD | CDKN2BAS | INTRON |
| rs7028268 | 37.1 | 9 | 22048414 | FORWARD | CDKN2BAS | INTRON |
| rs1537378 | 37.1 | 9 | 22061614 | FORWARD | CDKN2BAS | INTRON |
| rs4977574 | 37.1 | 9 | 22098574 | FORWARD | CDKN2BAS | INTRON |
| rs2383207 | 37.1 | 9 | 22115959 | FORWARD | CDKN2BAS | INTRON |
| rs1537376 | 37.1 | 9 | 22116220 | FORWARD | CDKN2BAS | INTRON |
| rs1333049 | 37.1 | 9 | 22125503 | FORWARD | CDKN2BAS/LOC729983 | INTERGENIC |
| rs10812610 | 37.1 | 9 | 27533984 | FORWARD | LOC100288294/C9orf72 | INTERGENIC |
| rs1888747 | 37.1 | 9 | 86155551 | FORWARD | C9orf103/FRMD3 | INTERGENIC |
| rs1875620 | 37.1 | 9 | 91540059 | FORWARD | C9orf47/LOC286238 | INTERGENIC |
| rs1883025 | 37.1 | 9 | 107664301 | REVERSE | ABCA1 | INTRON |
| rs10983013 | 37.1 | 9 | 118683862 | FORWARD | C9orf27 | INTRON |
| rs9408887 | 37.1 | 9 | 118713227 | FORWARD | PAPPA/C9orf27 | INTERGENIC |
| rs2519093 | 37.1 | 9 | 136141870 | FORWARD | ABO | INTRON |
| rs651007 | 37.1 | 9 | 136153875 | FORWARD | ABO/SURF6 | INTERGENIC |
| rs579459 | 37.1 | 9 | 136154168 | FORWARD | SURF6/ABO | INTERGENIC |
| rs649129 | 37.1 | 9 | 136154304 | REVERSE | ABO/SURF6 | INTERGENIC |
| rs635634 | 37.1 | 9 | 136155000 | REVERSE | ABO/SURF6 | INTERGENIC |
| rs2505083 | 37.1 | 10 | 30335122 | FORWARD | KIAA1462 | INTRON |
| rs7478408 | 37.1 | 10 | 44500807 | FORWARD | HNRNPA3P1/LOC100130539 | INTERGENIC |
| rs2047009 | 37.1 | 10 | 44539913 | FORWARD | LOC100130539/HNRNPA3P1 | INTERGENIC |
| rs2802493 | 37.1 | 10 | 44553583 | FORWARD | LOC100130539/HNRNPA3P1 | INTERGENIC |
| rs2818912 | 37.1 | 10 | 44583027 | FORWARD | HNRNPA3P1/LOC100130539 | INTERGENIC |
| rs501120 | 37.1 | 10 | 44753867 | REVERSE | HNRNPA3P1/LOC100130539 | INTERGENIC |
| rs1746048 | 37.1 | 10 | 44775824 | FORWARD | HNRNPA3P1/LOC100130539 | INTERGENIC |
| rs10761731 | 37.1 | 10 | 65027610 | FORWARD | JMJD1C | INTRON |
| rs1865020 | 37.1 | 10 | 78688976 | FORWARD | KCNMA1 | INTRON |
| rs11203032 | 37.1 | 10 | 90964614 | FORWARD | FAS/CH25H | INTERGENIC |
| rs11203042 | 37.1 | 10 | 90989109 | FORWARD | LIPA | INTRON |
| rs1412444 | 37.1 | 10 | 91002927 | REVERSE | LIPA | INTRON |
| rs2246833 | 37.1 | 10 | 91005854 | FORWARD | LIPA | INTRON |
| rs2068888 | 37.1 | 10 | 94839642 | FORWARD | LOC389997/CYP26A1 | INTERGENIC |
| rs4986893 | 37.1 | 10 | 96540410 | FORWARD | CYP2C19 | CODING |
| rs1799853 | 37.1 | 10 | 96702047 | FORWARD | CYP2C9 | CODING |
| rs1057910 | 37.1 | 10 | 96741053 | FORWARD | CYP2C9 | CODING |
| rs11597086 | 37.1 | 10 | 101953705 | FORWARD | CHUK | INTRON |
| rs12413409 | 37.1 | 10 | 104719096 | FORWARD | CNNM2 | INTRON |
| rs10509906 | 37.1 | 10 | 111757674 | FORWARD | XPNPEP1/ADD3 | INTERGENIC |
| rs1129555 | 37.1 | 10 | 113910721 | REVERSE | GPAM | UTR |
| rs2255141 | 37.1 | 10 | 113933886 | FORWARD | GPAM | INTRON |
| rs12296050 | 37.1 | 11 | 2489342 | FORWARD | KCNQ1 | INTRON |
| rs451041 | 37.1 | 11 | 3060725 | FORWARD | CARS | INTRON |
| rs2923084 | 37.1 | 11 | 10388782 | FORWARD | AMPD3/ADM | INTERGENIC |
| rs1471895 | 37.1 | 11 | 11341186 | FORWARD | GALNTL4 | INTRON |
| rs10128711 | 37.1 | 11 | 18632984 | FORWARD | SPTY2D1 | INTRON |
| rs11024739 | 37.1 | 11 | 18645843 | FORWARD | SPTY2D1 | INTRON |
| rs10832963 | 37.1 | 11 | 18664241 | FORWARD | TMEM86A/SPTY2D1 | INTERGENIC |
| rs11028255 | 37.1 | 11 | 24916152 | FORWARD | LUZP2 | INTRON |
| rs3136441 | 37.1 | 11 | 46743247 | FORWARD | F2 | INTRON |
| rs174546 | 37.1 | 11 | 61569830 | FORWARD | FADS1 | UTR |
| rs174550 | 37.1 | 11 | 61571478 | FORWARD | FADS1 | INTRON |
| rs174583 | 37.1 | 11 | 61609750 | FORWARD | FADS2 | INTRON |
| rs174601 | 37.1 | 11 | 61623140 | REVERSE | FADS2 | INTRON |
| rs11236530 | 37.1 | 11 | 75489404 | FORWARD | DGAT2 | INTRON |
| rs974819 | 37.1 | 11 | 103660567 | FORWARD | DYNC2H1/PDGFD | INTERGENIC |
| rs7937106 | 37.1 | 11 | 107354312 | FORWARD | ALKBH8/CWF19L2 | INTERGENIC |
| rs1558861 | 37.1 | 11 | 116607437 | FORWARD | LOC100288599/BUD13 | INTERGENIC |
| rs9326246 | 37.1 | 11 | 116611733 | FORWARD | LOC100288599/BUD13 | INTERGENIC |
| rs964184 | 37.1 | 11 | 116648917 | FORWARD | BUD13/ZNF259 | INTERGENIC |
| rs2266788 | 37.1 | 11 | 116660686 | REVERSE | APOA5 | UTR |
| rs7941030 | 37.1 | 11 | 122522375 | FORWARD | LOC100288752/LOC399959 | INTERGENIC |
| rs7115089 | 37.1 | 11 | 122530591 | FORWARD | UBASH3B | INTRON |
| rs563519 | 37.1 | 11 | 126028717 | FORWARD | CDON/RPUSD4 | INTERGENIC |
| rs11220462 | 37.1 | 11 | 126243952 | FORWARD | ST3GAL4 | INTRON |
| rs11220463 | 37.1 | 11 | 126248211 | FORWARD | ST3GAL4 | INTRON |
| rs11833579 | 37.1 | 12 | 775199 | FORWARD | LOC100132369/NINJ2 | INTERGENIC |
| rs1344454 | 37.1 | 12 | 8995211 | REVERSE | A2ML1 | INTRON |
| rs7973505 | 37.1 | 12 | 19164304 | FORWARD | PLEKHA5/CAPZA3 | INTERGENIC |
| rs11044389 | 37.1 | 12 | 19168581 | FORWARD | CAPZA3/PLEKHA5 | INTERGENIC |
| rs7979074 | 37.1 | 12 | 19169528 | FORWARD | PLEKHA5/CAPZA3 | INTERGENIC |
| rs1018298 | 37.1 | 12 | 19182772 | FORWARD | PLEKHA5/CAPZA3 | INTERGENIC |
| rs7134375 | 37.1 | 12 | 20473758 | FORWARD | LOC400013/PDE3A | INTERGENIC |
| rs4149056 | 37.1 | 12 | 21331549 | FORWARD | SLCO1B1 | CODING |
| rs2046383 | 37.1 | 12 | 30104142 | FORWARD | IPO8/TMTC1 | INTERGENIC |
| rs1520832 | 37.1 | 12 | 43253415 | REVERSE | ADAMTS20/PRICKLE1 | INTERGENIC |
| rs11613352 | 37.1 | 12 | 57792580 | FORWARD | INHBC/R3HDM2 | INTERGENIC |
| rs3741414 | 37.1 | 12 | 57844049 | REVERSE | INHBC | UTR |
| rs11172782 | 37.1 | 12 | 59259628 | FORWARD | XRCC6BP1/LRIG3 | INTERGENIC |
| rs17019682 | 37.1 | 12 | 92305270 | FORWARD | BTG1/DCN | INTERGENIC |
| rs10745954 | 37.1 | 12 | 103483094 | FORWARD | C12orf42/ASCL1 | INTERGENIC |
| rs7298565 | 37.1 | 12 | 109937534 | FORWARD | UBE3B | CODING |
| rs7134594 | 37.1 | 12 | 110000193 | FORWARD | MMAB | INTRON |
| rs3184504 | 37.1 | 12 | 111884608 | FORWARD | SH2B3 | CODING |
| rs648997 | 37.1 | 12 | 111976776 | REVERSE | ATXN2 | INTRON |
| rs11065987 | 37.1 | 12 | 112072424 | FORWARD | ATXN2/BRAP | INTERGENIC |
| rs11066001 | 37.1 | 12 | 112119171 | FORWARD | BRAP | INTRON |
| rs2238151 | 37.1 | 12 | 112211833 | FORWARD | ALDH2 | INTRON |
| rs671 | 37.1 | 12 | 112241766 | FORWARD | ALDH2 | CODING |
| rs17696736 | 37.1 | 12 | 112486818 | FORWARD | C12orf30 | INTRON |
| rs1169288 | 37.1 | 12 | 121416650 | REVERSE | LOC100287253/HNF1A | COMPLEX |
| rs1183910 | 37.1 | 12 | 121420807 | FORWARD | LOC100287253/HNF1A | INTERGENIC |
| rs74876709 | 37.1 | 12 | 121450013 | FORWARD | C12orf43 | INTRON |
| rs601339 | 37.1 | 12 | 123174743 | FORWARD | NIACR1/KNTC1 | INTERGENIC |
| rs4765127 | 37.1 | 12 | 124460167 | FORWARD | ZNF664 | INTRON |
| rs12310367 | 37.1 | 12 | 124486678 | FORWARD | ZNF664 | INTRON |
| rs838880 | 37.1 | 12 | 125261593 | FORWARD | NCOR2/UNKNOWN | INTERGENIC |
| rs9319428 | 37.1 | 13 | 28973621 | FORWARD | FLT1 | INTRON |
| rs9534275 | 37.1 | 13 | 32940345 | FORWARD | BRCA2 | INTRON |
| rs548097 | 37.1 | 13 | 75776268 | REVERSE | LOC100288208/LOC647288 | INTERGENIC |
| rs1041466 | 37.1 | 13 | 110244322 | FORWARD | MYO16/IRS2 | INTERGENIC |
| rs1411766 | 37.1 | 13 | 110252160 | REVERSE | IRS2/MYO16 | INTERGENIC |
| rs6492208 | 37.1 | 13 | 110257726 | FORWARD | MYO16/IRS2 | INTERGENIC |
| rs2391777 | 37.1 | 13 | 110258243 | FORWARD | IRS2/MYO16 | INTERGENIC |
| rs7989848 | 37.1 | 13 | 110283468 | FORWARD | IRS2/MYO16 | INTERGENIC |
| rs4773144 | 37.1 | 13 | 110960712 | FORWARD | COL4A2 | INTRON |
| rs9515203 | 37.1 | 13 | 111049623 | FORWARD | COL4A2 | INTRON |
| rs8017377 | 37.1 | 14 | 24883887 | FORWARD | KIAA1305 | CODING |
| rs12885474 | 37.1 | 14 | 25381377 | FORWARD | STXBP6 | INTRON |
| rs4903031 | 37.1 | 14 | 73019236 | FORWARD | RGS6 | INTRON |
| rs2895811 | 37.1 | 14 | 100133942 | FORWARD | HHIPL1 | INTRON |
| rs2412710 | 37.1 | 15 | 42683787 | FORWARD | CAPN3 | INTRON |
| rs2929282 | 37.1 | 15 | 44245931 | FORWARD | FRMD5 | INTRON |
| rs1532085 | 37.1 | 15 | 58683366 | FORWARD | AQP9/LIPC | INTERGENIC |
| rs261342 | 37.1 | 15 | 58731153 | REVERSE | LIPC | INTRON |
| rs340029 | 37.1 | 15 | 60894965 | FORWARD | RORA | INTRON |
| rs10519210 | 37.1 | 15 | 63737925 | FORWARD | CA12/USP3 | INTERGENIC |
| rs17228212 | 37.1 | 15 | 67458639 | FORWARD | SMAD3 | INTRON |
| rs2869036 | 37.1 | 15 | 78667572 | FORWARD | IREB2/CRABP1 | INTERGENIC |
| rs899997 | 37.1 | 15 | 79019578 | REVERSE | LOC646934 | UTR |
| rs1814880 | 37.1 | 15 | 79021140 | REVERSE | LOC646934 | INTRON |
| rs7177699 | 37.1 | 15 | 79089734 | FORWARD | ADAMTS7 | INTRON |
| rs4380028 | 37.1 | 15 | 79111093 | FORWARD | MORF4L1/ADAMTS7 | INTERGENIC |
| rs7173743 | 37.1 | 15 | 79141784 | FORWARD | ADAMTS7/MORF4L1 | INTERGENIC |
| rs17315707 | 37.1 | 15 | 80718699 | FORWARD | ARNT2 | INTRON |
| rs4778663 | 37.1 | 15 | 82346498 | FORWARD | MEX3B/EFTUD1 | INTERGENIC |
| rs17514846 | 37.1 | 15 | 91416550 | FORWARD | FURIN | INTRON |
| rs11649653 | 37.1 | 16 | 30918487 | FORWARD | CTF1/NCRNA00095 | INTERGENIC |
| rs10521222 | 37.1 | 16 | 51158710 | FORWARD | CYLD/SALL1 | INTERGENIC |
| rs1421085 | 37.1 | 16 | 53800954 | FORWARD | FTO | INTRON |
| rs9939609 | 37.1 | 16 | 53820527 | FORWARD | FTO | INTRON |
| rs7202116 | 37.1 | 16 | 53821615 | FORWARD | FTO | INTRON |
| rs247616 | 37.1 | 16 | 56989590 | FORWARD | CETP/HERPUD1 | INTERGENIC |
| rs3764261 | 37.1 | 16 | 56993324 | REVERSE | HERPUD1/CETP | INTERGENIC |
| rs7205804 | 37.1 | 16 | 57004889 | FORWARD | CETP | INTRON |
| rs1834018 | 37.1 | 16 | 58307242 | FORWARD | CCDC113 | INTRON |
| rs16942887 | 37.1 | 16 | 67928042 | FORWARD | PSKH1 | INTRON |
| rs2000999 | 37.1 | 16 | 72108093 | FORWARD | HPR | INTRON |
| rs7193343 | 37.1 | 16 | 73029160 | FORWARD | ZFHX3 | INTRON |
| rs12932445 | 37.1 | 16 | 73069888 | FORWARD | ZFHX3 | INTRON |
| rs2925979 | 37.1 | 16 | 81534790 | REVERSE | CMIP | INTRON |
| rs4284623 | 37.1 | 16 | 83556281 | FORWARD | CDH13 | INTRON |
| rs7187172 | 37.1 | 16 | 83561866 | FORWARD | CDH13 | INTRON |
| rs11640777 | 37.1 | 16 | 83562213 | FORWARD | CDH13 | INTRON |
| rs2070863 | 37.1 | 17 | 1648502 | FORWARD | SERPINF2 | CODING |
| rs2281727 | 37.1 | 17 | 2117945 | REVERSE | SMG6 | INTRON |
| rs216172 | 37.1 | 17 | 2126504 | REVERSE | SMG6 | INTRON |
| rs12936587 | 37.1 | 17 | 17543722 | FORWARD | RAI1/PEMT | INTERGENIC |
| rs881844 | 37.1 | 17 | 37810218 | REVERSE | STARD3 | INTRON |
| rs11869286 | 37.1 | 17 | 37813856 | FORWARD | STARD3 | INTRON |
| rs7225700 | 37.1 | 17 | 45391804 | FORWARD | C17orf57/ITGB3 | INTERGENIC |
| rs7206971 | 37.1 | 17 | 45425115 | FORWARD | C17orf57 | INTRON |
| rs46522 | 37.1 | 17 | 46988597 | FORWARD | UBE2Z | INTRON |
| rs1801689 | 37.1 | 17 | 64210580 | FORWARD | APOH | CODING |
| rs4148008 | 37.1 | 17 | 66875294 | REVERSE | ABCA8 | INTRON |
| rs4082919 | 37.1 | 17 | 76377482 | FORWARD | PGS1 | INTRON |
| rs4129767 | 37.1 | 17 | 76403984 | REVERSE | PGS1 | INTRON |
| rs7407640 | 37.1 | 18 | 12358997 | FORWARD | AFG3L2 | INTRON |
| rs7240405 | 37.1 | 18 | 47159090 | FORWARD | LIPG/ACAA2 | INTERGENIC |
| rs7239867 | 37.1 | 18 | 47164717 | FORWARD | LIPG/ACAA2 | INTERGENIC |
| rs17695069 | 37.1 | 18 | 56650936 | FORWARD | ZNF532 | INTRON |
| rs2168711 | 37.1 | 18 | 57848531 | REVERSE | MC4R/LOC728115 | INTERGENIC |
| rs17782313 | 37.1 | 18 | 57851097 | FORWARD | LOC728115/MC4R | INTERGENIC |
| rs11880198 | 37.1 | 19 | 3159769 | FORWARD | GNA15 | INTRON |
| rs8112883 | 37.1 | 19 | 7179320 | FORWARD | INSR | INTRON |
| rs2278236 | 37.1 | 19 | 8431581 | REVERSE | LOC100288364/ANGPTL4 | INTERGENIC |
| rs1122608 | 37.1 | 19 | 11163601 | FORWARD | LOC100287659 | INTRON |
| rs6511720 | 37.1 | 19 | 11202306 | FORWARD | LDLR | INTRON |
| rs2738459 | 37.1 | 19 | 11238473 | FORWARD | LDLR | INTRON |
| rs737337 | 37.1 | 19 | 11347493 | FORWARD | DOCK6 | CODING |
| rs10401969 | 37.1 | 19 | 19407718 | FORWARD | SF4 | INTRON |
| rs931608 | 37.1 | 19 | 22614122 | FORWARD | ZNF98/LOC100128139 | INTERGENIC |
| rs2075650 | 37.1 | 19 | 45395619 | FORWARD | TOMM40 | INTRON |
| rs7412 | 37.1 | 19 | 45412079 | FORWARD | LOC100129500 | CODING |
| rs439401 | 37.1 | 19 | 45414451 | FORWARD | LOC100129500/APOC1 | INTERGENIC |
| rs445925 | 37.1 | 19 | 45415640 | FORWARD | APOC1/LOC100129500 | INTERGENIC |
| rs4420638 | 37.1 | 19 | 45422946 | FORWARD | APOC1/LOC100129500 | INTERGENIC |
| rs492602 | 37.1 | 19 | 49206417 | REVERSE | FUT2 | CODING |
| rs386000 | 37.1 | 19 | 54792761 | FORWARD | LILRB2/LILRA3 | INTERGENIC |
| rs2208454 | 37.1 | 20 | 14265415 | FORWARD | FLRT3/MACROD2 | INTERGENIC |
| rs6029526 | 37.1 | 20 | 39672618 | FORWARD | TOP1/LOC100127886 | INTERGENIC |
| rs909802 | 37.1 | 20 | 39936815 | FORWARD | LPIN3/ZHX3 | INTERGENIC |
| rs8125304 | 37.1 | 20 | 41473735 | FORWARD | PTPRT | INTRON |
| rs1800961 | 37.1 | 20 | 43042364 | FORWARD | HNF4A | CODING |
| rs4810479 | 37.1 | 20 | 44545048 | FORWARD | FLJ40606/PLTP | INTERGENIC |
| rs6065906 | 37.1 | 20 | 44554015 | FORWARD | FLJ40606/PLTP | INTERGENIC |
| rs9982601 | 37.1 | 21 | 35599128 | FORWARD | C21orf82/KCNE2 | INTERGENIC |
| rs2836878 | 37.1 | 21 | 40465534 | FORWARD | PSMG1/LOC100289305 | INTERGENIC |
| rs181362 | 37.1 | 22 | 21932068 | REVERSE | UBE2L3 | INTRON |
| rs2284038 | 37.1 | 22 | 37635055 | FORWARD | RAC2 | INTRON |
| rs5756931 | 37.1 | 22 | 38546033 | FORWARD | PLA2G6 | INTRON |
| rs28371725 | 37.1 | 22 | 42523805 | FORWARD | CYP2D6 | INTRON |
| rs5030656 | 37.1 | 22 | 42524176 | FORWARD | CYP2D6 | CODING |

**Suppplementary** **Table 2:** Baseline characteristics of LIPID Genetic Cohort at entry to the LIPID trial

| LIPID Genetic Cohort (All) | 4,932 (100%) |
| --- | --- |
| Pravastatin | 2,524 (51.2%) |
| Age (years) | 60.2 (8.41) |
| Male | 4,145 (84.0%) |
| Hypertension | 2,073 (42.0%) |
| Diabetes | 352 (7.1%) |
| Body mass index, kg/m^2^ | 26.7 (3.75) |
| Total cholesterol, mmol/L | 5.65 (0.82) |
| HDL cholesterol, mmol/L | 0.95 (0.23) |
| Triglycerides, mmol/L | 20 (0.4%) |
| Current smoker | 427 (8.7%) |
| History of stroke | 190 (3.9%) |
| Qualifying event myocardial infarction | 3,218 (65.2%) |
| Angina grade > 0 | 1,637 (33.2%) |
| Dyspnea NYHA grade > 1 | 387 (7.8%) |
| White blood count (10^9^/L) | 7.15 (1.75) |
| Systolic blood pressure (mmHg) | 134 (19.1) |
| Atrial fibrillation | 49 (1.0%) |
| Aspirin | 4,136 (83.9%) |
| Peripheral vascular disease | 473 (9.6%) |

**LIPID Study Group Management Committee**—A Tonkin (chair), D Colquhoun, P Glasziou, D Hunt, A Keech, P Nestel, J Simes, P Thompson, D Sullivan, R Stewart, M West, H White. NHMRC Clinical Trials Centre, University of Sydney—E Barnes, W Hague, A Keech, A Kirby, K Mann, H Pater, R Pike, J Simes.

**LIPID Investigators**—I Beinart, H McKee (Albury, New South Wales, Australia); R Abraham, G Parnell (Blacktown, NSW); J England, A Viles (Blue Mountains, NSW); N Campbell, S Grant (Bowral, NSW); M O’Neill, R Wikramanayake (Canterbury, NSW); J Crowe, J Waites (Coffs Harbour, NSW); R Portley, R Wyndham (Concord, NSW); J Pallas, J Woods (Gosford, NSW); K Hellestrand, B Harvey (Hornsby, NSW); D Owensby, J Ryan (Illawarra, NSW); J Silberberg, M Taylor (John Hunter, NSW); E Breed, K Wee (Kempsey, NSW); A McLean, K Quinn (Nepean, NSW); A Russell, W Walsh (Prince Henry, NSW); G Nelson, J Padley (Royal North Shore, NSW); P Harris, M Threlfall (Royal Prince Alfred, NSW); D Ramsay, J Rubendra (St George, NSW); T Campbell, S D’Arcy (St Vincent’s, NSW); B Cuthbert, N Cuthbert (Tweed Heads, NSW); M Neaverson, M Russell (Western Suburbs, NSW); D McGill, P Taverner (Woden Valley, NSW); H Briggs, A Broughton (Alfred, Victoria); L Brown, A Tonkin (AMRC Austin, Victoria); A Driscoll, A Hamer (AMRC Heidelberg, Victoria); Y Cavenett, W Ryan (Box Hill, Victoria); J Counsell, M Martin (Dandenong, Victoria); I Lyall, B Tyack (Geelong, Victoria); H Harrap, R Ziffer (Gippsland, Victoria); A Buncle, B Feldtmann (Goulburn Valley, Victoria); M Burggraaf, C Winter (Latrobe, Victoria); K Barnett, D Rose (Maroondah, Victoria); A Soward, L Morgan (Mildura, Victoria); G Savige, M Wahlqvist (Monash, Victoria); B Jackson, G Rudge (Northern, Victoria); D Hunt, M Sallaberger (Royal Melbourne, Victoria); T Howison, J McCabe (Wimmera, Victoria); S Hodgens, C Medley (Wodonga, Victoria); T Carruthers, B Cooke (Cairns, Queensland); G Aroney, P Hicks (Gold Coast, Queensland); D Careless, H LeGood (Ipswich, Queensland); K Roberts, J Sampson (Logan, Queensland); F Ekin, G Real (Maryborough, Queensland); L Ross-Lee, S Woodhouse (Mater Private, Queensland); S Coverdale, V Smith-Orr (Nambour, Queensland); B Wicks, J Wicks (Pindara, Queensland); A Carle, M West (Prince Charles, Queensland); P Carroll, D Chaseling (Redcliffe, Queensland); D Colquhoun, B Gallagher (Wesley, Queensland); B Currie, D Kane (Rockhampton, Queensland); C Atkinson, R Bradfield (Royal Brisbane, Queensland); T Htut, L Hughes (Toowoomba, Queensland); K Gunawardane, A Heyworth (Townsville, Queensland); P Aylward, F Waters (Flinders, South Australia); L Callaway, R McLeay (Port Lincoln, South Australia); R Prideaux, Y Zhang (Queen Elizabeth, South Australia); I Hamilton- Craig, S Mackintosh (Repatriation General, South Australia); J Bradley, N Ely (Royal Adelaide, South Australia); G Lane, G Tulloch (Fremantle, Western Australia); R Burton, R Taylor (Royal Perth, Western Australia); J Hargan, P Thompson (Sir Charles Gairdner, Western Australia); W Hitchener, B Singh (Launceston, Tasmania); M Smith, M Templer (North West Regional, Tasmania); P Neid, A Thomson (Royal Hobart, Tasmania); T Cook, M Gluyas (Ashburton, New Zealand); R Ronaldson, N Sharpe (Auckland); H Ikram, T Lawson (Christchurch); D Scott, R Stewart (Dunedin); D Clarke, J Reddy (Gisborne); M Denton, H White (Green Lane); J Kenyon, R Luke (Hastings); A Cuthbert, S Mann (Hutt); R Coxon, J Scott (Middlemore); P Foster-Pratt, R Luke (Napier); P Barclay, D Fry (Nelson); H Hart, P Wright (North Shore); J Calton, R Rankin (Northland); S Reuben, P Yorke (Southland); R Anandaraja, S Anandaraja (Taranaki); J Bruning, L Nairn (Tauranga); D Roy, H Roy (Timaru); D Friedlander, E Low (Waikato); P Healy, J Hedley (Wairau); P Heuser, P Leslie (Wellington).
